# Perceived Discrimination at School and Developmental Outcomes among Bai Adolescents: The Mediating Roles of Self-Esteem and Ethnic Identity

**DOI:** 10.3390/ijerph19020657

**Published:** 2022-01-07

**Authors:** Lifen Zhao, Steven Sek-yum Ngai

**Affiliations:** Department of Social Work, Chinese University of Hong Kong, Hong Kong 999077, China; syngai@cuhk.edu.hk

**Keywords:** developmental outcomes, perceived academic discrimination, perceived ethnic discrimination, self-esteem, ethnic identity, ethnic minority adolescents, China

## Abstract

Although discrimination is widely acknowledged to impair developmental outcomes among ethnic minority adolescents, literature differentiating discrimination based on personal characteristics and group membership is lacking, especially in Chinese contexts, and the mechanisms of those relationships remain unclear. In response, the study presented here examined whether self-esteem mediates the relationship between perceived academic discrimination and developmental outcomes among such ethnic minority adolescents, and whether ethnic identity mediates the relationship between perceived ethnic discrimination and developmental outcomes. Multistage cluster random sampling performed in Dali and Kunming, China, yielded a sample of 813 Bai adolescents whose data was analysed in structural equation modelling. The results indicate that perceived academic discrimination had a direct negative effect on adolescents’ mental health, while perceived ethnic discrimination had direct negative effects on their behavioural adjustment and social competence. Perceived academic discrimination also indirectly affected adolescents’ behavioural adjustment, mental health, and social competence via self-esteem, whereas perceived ethnic discrimination indirectly affected their behavioural adjustment and social competence via ethnic identity. These findings deepen current understandings of how perceived discrimination, self-esteem, and ethnic identity affect the developmental outcomes of ethnic minority adolescents and provide practical recommendations for policymakers and social workers to promote those outcomes in China.

## 1. Introduction

Discrimination refers to any behaviour that denies individuals or social groups equal treatment [1,2]. Experiences of discrimination and/or unfair treatment have been characterised as part of everyday life among ethnic minority adolescents [3], especially at school [4,5], where adolescents generally spend a great deal of their time [6]. In turn, their perceived discrimination can contribute to adverse developmental outcomes, including worse mental health, negative behaviours, and weak social competence [7,8,9]. In mainland China, a multi-ethnic nation of 56 officially recognised ethnic groups, including the Han majority group and 55 ethnic minority groups, there are more than 19.9 million ethnic minority adolescents aged 10–20 years old, which accounts for approximately 9.81% of the entire population in that age range [10]. However, studies on the relationships between such adolescents’ perceived discrimination at school and their developmental outcomes have rarely been conducted in mainland China.

As attested in the literature, reasons for being discriminated against can vary widely. Some researchers in Western contexts have classified discrimination into two types: discrimination based on personal characteristics and discrimination based on group membership [11]. Whereas discrimination based on personal characteristics occurs when a person is discriminated due to their personal traits (e.g., age and appearance), discrimination based on group membership occurs on the basis of person’s belonging to a particular social group—for instance, an ethnic group [12]. Nevertheless, studies on the relationship between perceived discrimination and ethnic minority adolescents’ developmental outcomes in mainland China have rarely differentiated discrimination based on personal characteristics from discrimination based on group membership. Thus, given the prevalence of academic discrimination in China [13] and the fact that ethnic discrimination is a unique negative experience for ethnic minorities, it is necessary to empirically investigate their effects as kinds of discrimination based on personal characteristics and group membership, respectively, on the developmental outcomes of ethnic minority adolescents in mainland China.

As suggested by symbolic interactionist theory, self-esteem, meaning one’s feelings of self-worth and self-respect [14], may mediate the relationship between adolescents’ negative experiences and developmental outcomes [15,16,17]. In particular, if adolescents are discriminated against, then they may internalise others’ negative appraisals of them and develop a negative self-concept, which may adversely affect their developmental outcomes. At the same time, social identity theory indicates that ethnic identity, defined as the part of an individual’s self-concept deriving from membership in an ethnic group together with that membership’s value and emotional significance [18,19], may mediate the relationship between ethnic minority adolescents’ ethnic discrimination and developmental outcomes [20]. Despite theories shedding light on the mechanisms of perceived academic discrimination and ethnic discrimination on adolescents’ developmental outcomes, empirical studies testing potential mediators in those relationships (e.g., self-esteem and ethnic identity) have been few, especially among ethnic minority adolescents in mainland China.

Against that background, we sought to narrow those gaps in the literature by examining the direct effect of perceived academic discrimination and ethnic discrimination on developmental outcomes, the potential mediating effect of self-esteem between perceived academic discrimination and those outcomes, and the potential mediating effect of ethnic identity between perceived ethnic discrimination and the outcomes among ethnic minority adolescents in mainland China.

### 1.1. Perceived Discrimination and Developmental Outcomes

According to Erikson’s theory of psychosocial development [21,22], people experience different life crises and developmental tasks at different stages of life, and successfully resolving those crises results in healthy development. During adolescence, for example, individuals are expected to develop a stable personality, prepare themselves for culturally acceptable adult roles and responsibilities, and learn the mechanisms of interpersonal adult relationships [23,24]. Accordingly, behavioural adjustment and mental health have been widely used to measure adolescents’ developmental outcomes [25,26]. Beyond that, owing to the developmental task of learning the mechanisms of interpersonal relationships, social competence is regarded as a vital dimension of adolescents’ development. For that reason, mental health, behavioural adjustment, and social competence served as indicators of developmental outcomes in our study.

Theoretical studies have validated perceived discrimination’s detrimental effects on the developmental outcomes of ethnic minority adolescents. The integrative model developed by Coll et al. [27] casts valuable light on the relationship between ethnic minority adolescents’ perceived discrimination and their developmental outcomes. Taking an ecological approach, the model stresses that such adolescents’ development should be considered in terms of the environmental practices of racism, prejudice, and discrimination that factor into their development [28,29].

The education system in mainland China regards academic achievement as the primary, if not the sole, criterion for assessing whether adolescents are excellent. As a consequence, students who demonstrate poor academic achievement are more likely to be discriminated against [30]. In turn, academic discrimination among adolescents is negatively associated with their development. In past work, adolescents who had experienced academic discrimination were more likely to have more stress, less self-confidence, and worse mental health [31,32]. In another study, Zu [33] showed that academic discrimination could increase anxiety among students with poor academic achievement, as well as discourage their social interaction. In addition, Jia [34] has suggested that students who have been insulted, devaluated, or threatened because of their academic achievement tend to have higher levels of anxiety and exhibit more aggressive behaviours. Although research has validated perceived academic discrimination’s negative association with adolescents’ development, few empirical studies have investigated that relationship among ethnic minority adolescents, despite their tendency to demonstrate poor academic achievement [35]. In response, our study explored the relationship between perceived academic discrimination and developmental outcomes among ethnic minority adolescents in mainland China.

In the past two decades, multiple empirical studies have validated perceived ethnic discrimination’s negative association with adolescents’ developmental outcomes [36,37]. Discrimination can pose a significant risk to the mental health of ethnic minority adolescents, and experiences of discrimination have been shown to have negative consequences for their psychological functioning, including elevated anxiety, stress, and depressive symptoms [38,39,40], and feelings of loneliness [41]. Experience with discrimination is also a contributing factor to poor behavioural outcomes [42], and in longitudinal research has been prospectively associated with more violent behaviours [43]. Similar findings emerged in the study of McKenney et al. [44], which showed that experiences with ethnic discrimination triggered ethnic minority adolescents’ engagement in aggressive and violent behaviours.

Although studies have addressed the relationship between perceived discrimination and both psychological and behavioural outcomes, very few have addressed the potential relationship between perceived discrimination and social competence. A notable exception is the research conducted by Myrick et al. [36], which revealed that perceived discrimination was negatively related to social competence. Even so, most studies on the relationship between ethnic discrimination and developmental outcomes have been conducted in Western contexts, whereas few researchers have examined such discrimination’s effect on the developmental outcomes of ethnic minority adolescents in mainland China. Therefore, it is necessary to examine the specific relationship between ethnic discrimination and developmental outcomes in that population.

### 1.2. Self-Esteem and Ethnic Identity

Social identity theory distinguishes personal identity from social identity. Personal identity refers to an individual’s self-concept, which is more personal in nature and usually denotes their specific attributes (e.g., physical features, psychological characteristics, and feelings of self-worth), whereas social identity denotes the part of an individual’s self-concept, which derives from their knowledge of their membership in a social group (or groups) along with the value and emotional significance attached to that membership [18,45]. Although personal identity and social identity are parts of the self, they refer to different, equally authentic levels of self-concept that cannot be reduced to one another [46,47]. After all, people are not only individuals but also members of social groups, and self-concept at the personal and group levels is equally real [48].

Aside from differentiating personal identity from social identity, social identity theory recognises a functional antagonism between those two levels of identity in terms of their salience. It maintains that because the functioning of identity depends upon the situation, particular identities tend to be activated in particular situations [18,47]. Verifying that view, empirical research has shown that discrimination based on personal characteristics is associated with self-concept at the personal level but not ethnic identity, and the reverse is true for ethnic discrimination [48].

Because we examined academic discrimination, which is based on personal characteristics, and ethnic discrimination, which is based on social group, self-concept at both the personal and social levels was examined as well. At the same time, the study also investigated feelings of self-worth as a vital part of personal identity called self-esteem and ethnic identity as a kind of social identity. Considering that particular identities tend to be activated in particular situations, academic discrimination likely relates to self-esteem, whereas ethnic discrimination likely relates to ethnic identity.

### 1.3. Self-Esteem as a Mediator

According to symbolic interactionist theory, one’s self-concept is primarily established by interacting with other people. Because individuals rely upon feedback from others to establish their self-concept, perceptions of discrimination may convince them to accept others’ negative appraisals of them, and, as a consequence, develop a lower level of self-worth [16,49]. Thus, if adolescents are discriminated against due to their academic performance, then they may internalise others’ negative appraisals or stereotypes and develop low levels of self-esteem, which can affect their developmental outcomes.

Studies have revealed not only the negative relationship between perceived academic discrimination and adolescents’ self-esteem [50,51] but also the associations between self-esteem and adolescents’ developmental outcomes, including behavioural adjustment and mental health [52,53,54]. In particular, adolescents with higher levels of self-esteem have exhibited more prosocial behaviours [55], less risky behaviours [56,57], and less negative psychological outcomes, such as depression [58,59]. Research has additionally highlighted the mediating role of self-esteem between negative experiences (e.g., social exclusion and psychological maltreatment) and developmental outcomes [60,61]—that is, that an individual’s self-esteem positively contributes to their coping with stressors and, in turn, their developmental outcomes. Following that logic, self-esteem may mediate adolescents’ perceived academic discrimination and developmental outcomes. However, to the best of our knowledge, empirical studies have rarely investigated self-esteem’s mediating role in that relationship, especially among ethnic minority adolescents in Chinese contexts.

### 1.4. Ethnic Identity as a Mediator

Social identity theory may shed light on ethnic identity’s mediating effect in the relationship between perceived ethnic discrimination and developmental outcomes among adolescents. According to the theory, when individuals identify with their social group (e.g., ethnic group), they focus on the group’s positive aspects and are proud of and self-confident about their membership in the group [62,63]. Experiencing discrimination from out-group members only intensifies their identification with their social group, which alleviates some of the harm done to their well-being and developmental outcomes. Therefore, experiencing ethnic discrimination may in fact enhance ethnic identity, and, in turn, decrease its negative effects on developmental outcomes.

Beyond theory, empirical studies have verified the mediating role of identification with one’s social group in the relationship between perceived discrimination and developmental outcomes [20,64,65]. However, other research has shown that ethnic identity mediates the discrimination–distress relationship among men but not women [66]. Findings regarding the effect of perceived ethnic discrimination on ethnic identity have also been mixed. For example, Pahl and Way [67] have validated social identity theory by showing that discrimination prompts the search for belonging and attachment in a marginalised group. Those findings suggest that experiencing discrimination from out-group members can intensify individuals’ identification with their group [68,69,70]. However, ethnic minorities may also be determined to gain acceptance from the majority and thus downplay their ethnicity [71]. In such cases, perceived ethnic discrimination negatively affects individuals’ ethnic identity [48]. Despite limited studies having been conducted in Chinese contexts, the existing two studies both have indicated that perceived ethnic discrimination reduces one’s identification with ethnic groups [72,73], which seems to suggest that ethnic discrimination contributes to a negative reconstruction of ethnic identity in Chinese culture.

All of those inconsistent findings indicate that the mediating effect of ethnic identity in the relationship between perceived ethnic discrimination and developmental outcomes needs to be further examined. In addition to that, recent studies on the mental health of adolescents have failed to examine adolescents’ other developmental outcomes, including behavioural adjustment and social competence. Because so few of those studies have been conducted in China, more research is warranted that explores ethnic identity’s mediating effect in the relationship between perceived ethnic discrimination and various developmental outcomes among ethnic minority adolescents in China.

### 1.5. The Present Study

**Figure 1:**
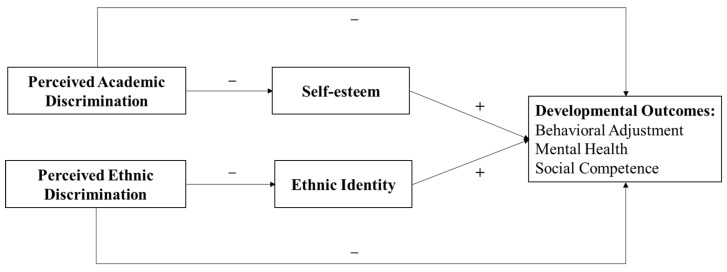
Theoretical framework and research hypotheses.

As the literature review has shown, despite well-established evidence of the correlation between perceived discrimination and developmental outcomes among adolescents, research differentiating discrimination based on personal characteristics versus group membership has been few and far between, especially in the population of ethnic minority adolescents in Chinese contexts. On top of that, although the association between ethnic discrimination and ethnic identity has been widely explored, the conclusions drawn have been inconsistent. Last, the potential mediating role of self-esteem in the relationships between perceived academic discrimination and developmental outcomes, and the potential mediator of ethnic identity in the relationships between perceived ethnic discrimination and the same outcomes has yet to be tested in an integrated framework. In response, our study was designed to address those gaps in the research. Figure 1 illustrates the study’s conceptual framework, from which four hypotheses were developed:

**Hypothesis** **1.** *Adolescents with a higher level of perceived academic discrimination are more likely to have lower levels of behavioural adjustment, mental health, and social competence.*

**Hypothesis** **2.** *Adolescents with a higher level of perceived ethnic discrimination are more likely to have lower levels of behavioural adjustment, mental health, and social competence.*

**Hypothesis** **3.** *Adolescents with a higher level of perceived academic discrimination are more likely to have less self-esteem, which lowers their levels of behavioural adjustment, mental health, and social competence.*

**Hypothesis** **4.** *Adolescents with a higher level of perceived ethnic discrimination are more likely to have a lower level of ethnic identity, which lowers their levels of behavioural adjustment, mental health, and social competence.*

## 2. Materials and Methods

Considering the fact that key variables of this study (e.g., ethnic identity and social competence) are complex phenomena that can neither be observed directly nor measured accurately with one single item, and the complex relationships among variables within the theoretical framework, structural equation modelling was performed to test the research hypotheses. This statistical analysis procedure provides significant advantages in examining relationships among latent variables measured by multiple items, and allows simultaneous tests of all the relationships [74], which has been widely used for testing mediation models [75,76]. The following paragraphs describe the participants and procedure, measures, and data analysis strategy.

### 2.1. Participants and Procedure

Following a procedure of multistage cluster random sampling, a self-administrated survey was conducted from October to December in 2019, in the cities of Dali and Kunming in Yunnan Province, China, which is home to numerous ethnic minority groups [73]. Once four districts were selected from the cities—two in Dali, two in Kunming—four middle schools, one from each district, were chosen at random. The four selected schools all agreed to participate in the survey. In each grade, four classes were randomly selected, and all students in those classes were invited to participate. Informed consent was obtained from all invited participants and their parents prior to the study, which was approved by our affiliated institution’s Survey and Behavioural Research Ethics Committee. Ultimately, 813 Bai adolescents were recruited; their demographic information appears in Table 1.

Bai is a large ethnic group in Yunnan province. Despite more and more similarities being found between Bai and Han people owing to their increasing communications, the differences in language, religious beliefs, customs and traditions distinguish Bai from Han people in China. While keeping their unique cultures and traditions, Bai people adopt new cultural elements to enrich their original culture, resulting in their connection to the Bai community as well as to the mainstream culture [77].

### 2.2. Measures

#### 2.2.1. Developmental Outcomes

Developmental outcomes were measured in three dimensions: behavioural adjustment, mental health, and social competence. First, behavioural adjustment was measured using three items (e.g., “Assisting schools and social service organisations to carry out activities”) adapted from the Behavioural Adjustment Scale [78], and were rated on a 5-point Likert scale ranging from 1 (strongly disagree) to 5 (strongly agree). The composite score of the three items yielded a reliability alpha coefficient of 0.665. Second, mental health was assessed using three items (e.g., “I feel lonely”) adapted from the Mental Health Scale [79] and rated on a 5-point Likert scale ranging from 1 (strongly disagree) to 5 (strongly agree). The reliability of the scale was 0.772. Last, social competence was measured using five items (e.g., “I know how to communicate with others”) adapted from the Social Competence subscale of the Chinese Positive Youth Development Scale [80], rated on a 5-point Likert scale ranging from 1 (strongly disagree) to 5 (strongly agree). The scale achieved a reliability alpha coefficient of 0.791.

#### 2.2.2. Self-Esteem

Self-esteem was measured using four items (e.g., “I am able to do things as well as most other people”) adapted from the Rosenberg Self-Esteem Scale [14]. Although the original response scale ranged from 1 (strongly disagree) to 4 (strongly agree), it was expanded to a 5-point scale to be consistent with other instruments used in the study. The scale had a reliability alpha coefficient of 0.801.

#### 2.2.3. Ethnic Identity

Ethnic identity was measured using four items (e.g., “I understand pretty well what my ethnic group membership means to me”) adapted from Phinney and Ong’s [81] Multigroup Ethnic Identity Measure-Revised scale and rated on a 5-point Likert scale ranging from 1 (strongly disagree) to 5 (strongly agree). The scale had a reliability alpha coefficient of 0.774.

#### 2.2.4. Perceived Academic Discrimination

Perceived academic discrimination was measured using three items (e.g., “I was wrongly disciplined or given after-school detention”) from the Educational Discrimination Distress subscale of the Adolescent Discrimination Distress Index [82]. On a 5-point Likert scale ranging from 1 (never) to 5 (always), participants indicated how often they had experienced discrimination due to their academic achievement. The composite score of the three items yielded a reliability alpha coefficient of 0.622.

#### 2.2.5. Perceived Ethnic Discrimination

Perceived ethnic discrimination was assessed using three items (e.g., “People act as if I am not smart”) adapted from the Everyday Discrimination Scale [83]. Participants were asked to indicate how often they had experienced discrimination due to their ethnicity on a 5-point Likert scale ranging from 1 (never) to 5 (always). The composite score of the three items yielded a reliability alpha coefficient of 0.798.

#### 2.2.6. Covariates

Sociodemographic variables, including age, gender (1 = male, 0 = female), household socioeconomic status, and school type (1 = ethnic minority school, 0 = ordinary school), were controlled when testing the hypotheses. Considering the possible unreliability of household income [84], household socio-economic status was measured with two indicators: parents’ level of education (1 = less than primary school, 6 = university or more) and parents’ employment status (1 = unemployed, 5 = senior managers) following Shi and Shen [85].

### 2.3. Data Analysis Strategy

To test the relationships between the independent variables, dependent variables, and mediating variables, structural equation modelling was performed in Amos version 25.0. First, a measurement model was tested in confirmatory factor analysis to examine how well the observed variables represented the corresponding latent variables. After the measurement model was validated, the structural paths among the key variables were tested in a structural model. Three indicators of goodness-of-fit were adopted to assess both the measurement and structural models. The first was the chi-square value (χ2), for which a non-significant χ2 indicates a good model fit [86]. Considering that χ2 is sensitive to sample size [87], a significant χ2 is also acceptable when the sample size is large (*N* > 200) [88]. The second was the comparative fit index (CFI), for which values exceeding 0.90 generally indicate a good fit [89]. The third was the root mean square error of approximation (RMSEA), for which values less than 0.08 indicate a “close fit” [88]. Last, mediating effects were tested by bootstrapping with 2000 iterations and bias-corrected 95% confidence intervals (CI) [90]. Any indirect effect with a CI excluding 0 indicated a significant mediating effect on the dependent variables.

## 3. Results

### 3.1. Descriptive Statistics and Preliminary Analyses

Table 2 provides a correlation matrix summarising the bivariate correlations between the key variables. As shown, most of the variables were significantly associated with each other in the expected directions. Perceived academic discrimination was negatively correlated with self-esteem and the three dimensions of the developmental outcomes (i.e., behavioural adjustment, mental health, and social competence). Meanwhile, perceived ethnic discrimination was negatively correlated with self-esteem, ethnic identity, and the three dimensions of the developmental outcomes, and both self-esteem and ethnic identity were positively related with all three dimensions as well.

### 3.2. Test of the Measurement Model

The measurement model showed a good fit (χ2 = 495.759, *df* = 254, *p* < 0.001, CFI = 0.961, and RMSEA = 0.034), and all observed variables were significantly loaded on the corresponding latent constructs. The standardised factor loadings of indicators for each latent construct in the measurement model ranged from 0.372 to 0.828, and thus met the commonly adopted threshold for acceptable loadings (0.30) [91], which suggests that the indicators represented the underlying constructs in a statistically reliable manner. Table 3 presents the standardised factor loadings of all indicators on each latent construct.

### 3.3. Test of the Structural Model

**Figure 2:**
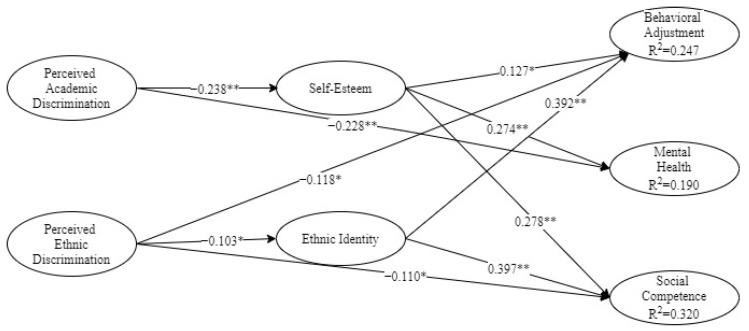
Standardised solutions for the structural model. Note. * *p* < 0.05, ** *p* < 0.01.

Figure 2 demonstrates the standardised solutions for the structural model; for brevity’s sake, only significant paths are displayed. In addition, the total, direct, and indirect effects generated from bootstrapping are presented in Table 4. The structural model provided a good fit with the data (χ2 = 807.244, *df* = 340, *p* < 0.001, CFI = 0.928, RMSEA = 0.041). In all, the model explained 24.7% of the variance for behavioural adjustment, 19.0% for mental health, and 32.0% for social competence.

The results indicate that perceived academic discrimination was directly related to mental health (*β* = −0.228, *p* < 0.01) but not behavioural adjustment or social competence. It was also indirectly related to behavioural adjustment (*β* = −0.030, *p* <0.05), mental health (*β* = −0.065, *p* < 0.001), and social competence (*β* = −0.066, *p* < 0.001). Those results suggest that self-esteem partly mediated the relationship between perceived academic discrimination and mental health, and fully mediated the relationships between perceived academic discrimination and both behavioural adjustment and social competence.

Meanwhile, perceived ethnic discrimination was directly associated with behavioural adjustment (*β* = −0.118, *p* < 0.05) and social competence (*β* = −0.110, *p* < 0.05) but not mental health. It was also indirectly associated with behavioural adjustment (*β* = −0.040, *p* < 0.05) and social competence (*β* = −0.041, *p* < 0.05) but not mental health. Those results indicate that ethnic identity partly mediated the relationship between perceived ethnic discrimination and adolescents’ behavioural adjustment and social competence.

## 4. Discussion

Although research on perceived discrimination and developmental outcomes has advanced greatly during the past decade, lingering gaps in knowledge on the topic need to be filled. Thus, in a sample of ethnic minority adolescents in mainland China, our study examined the correlation between perceived discrimination and developmental outcomes, self-esteem’s mediating role in the association between perceived academic discrimination and those outcomes, and ethnic identity’s mediating role in the association between perceived ethnic discrimination and the outcomes.

Among the results, whereas perceived academic discrimination was negatively associated with mental health, perceived ethnic discrimination was negatively associated with behavioural adjustment and social competence. Those findings are consistent with the integrative model developed by Coll et al. [27], and the results of previous empirical studies [34,92] showing that perceived discrimination contributes to poor developmental outcomes among ethnic minority adolescents. In other words, adolescents who have been discriminated against tend to have worse behavioural outcomes and social competence. However, perceived discrimination was not associated with every dimension of the developmental outcomes. An explanation for this discrepancy could be that studies have usually examined only one kind of discrimination or one dimension of developmental outcomes [93,94]. However, in our study, perceived discrimination demonstrated different effects on development when two kinds of discrimination (i.e., perceived academic discrimination and perceived ethnic discrimination) and three dimensions of developmental outcomes (i.e., behavioural adjustment, mental health, and social competence) were considered at once.

Our study also revealed that self-esteem functioned as a mediator in the associations between perceived academic discrimination and all three of those dimensions. Perceived academic discrimination was significantly associated with lower self-esteem, which lowered levels of the developmental outcomes among the adolescents. Those findings maintain symbolic interactionist theory and corroborate the results of previous empirical studies [16,51], which indicate that perceived academic discrimination can cause adolescents to accept others’ negative appraisals of them and develop low self-esteem. However, whereas previous research on the topic has primarily targeted ethnic majority adolescents—that is, Han adolescents—our research has extended those studies by revealing that perceived academic discrimination significantly reduced the self-esteem of ethnic minority adolescents.

Furthermore, self-esteem positively affected all the three dimensions of adolescents’ developmental outcomes in our study. This result aligns with previous results showing that self-esteem was associated with multiple positive developmental outcomes [55,95,96]. Self-esteem has been recognised as an important personal resource for promoting developmental outcomes among adolescents and as an important factor in mitigating social maladjustment [97], which suggests that self-esteem is positively associated with developmental outcomes.

Among our other results, perceived ethnic discrimination negatively affected ethnic identity, which consequently predicted adolescents’ behavioural adjustment and social competence. This result partly supports social identity theory, which holds that experiencing ethnic discrimination from out-group members intensifies individuals’ identification with their ethnic group, which serves to alleviate some of the harm done to their developmental outcomes [63]. Consistent with that theory, our study showed that ethnic identity mediated the association between perceived ethnic discrimination and adolescents’ developmental outcomes. However, instead of the positive relationship proposed by social identity theory, a negative relationship emerged between perceived ethnic discrimination and ethnic identity, as also found in a previous study conducted in a Chinese context [73].

A possible reason for that conflicting result is that considerable differences exist between ethnic minorities in Western countries and ones in mainland China, due to their different social contexts. In Western contexts, on the one hand, ethnic minority groups are from different countries and have distinct skin colours, languages, and customs. Even though societies in those contexts prohibit ethnic discrimination, such discrimination remains prevalent [98]. In such cases, group boundaries are considered to be impermeable, and status relations are considered to be stable. To maintain a positive identity, individuals in those contexts are likely to turn to their own ethnic groups, which can provide them with a sense of belonging and, in turn, strengthen their ethnic identity [99]. However, in Chinese contexts, although ethnic minorities have their own languages and customs, they share the Chinese culture in which they are born and live. Education in patriotism, ethnic equality, and unity are emphasised from primary education onwards, and various supporting policies for ethnic minorities have been implemented. As a result, group boundaries are considered to be permeable. When discriminated against, individuals are more likely to pursue personal goals to maintain their positive identity instead of turning to their ethnic group [100]. Under those circumstances, perceived ethnic discrimination may threaten instead of enhance one’s ethnic identity. In that way, studies conducted in different social contexts may suggest different conclusions. Such differences capture the inappropriateness of directly imposing Western theories in non-Western contexts, at least Chinese ones, and suggest that cross-cultural validity should be tested with empirical data.

Another finding was ethnic identity’s positive association with behavioural adjustment and social competence, which corroborates past results suggesting that ethnic identity predicts positive developmental outcomes [64,65,101,102]. However, ethnic identity had no significant effect on adolescents’ mental health, which conflicts with what previous research has shown [103]. A possible reason for this inconsistency could be that researchers have usually considered only ethnic identity as a variable of self-concept [104]. However, in our study, self-concept on both the personal level (i.e., self-esteem) and social level (i.e., ethnic identity) were included, and self-esteem was a strong predictor of adolescents’ mental health. Accordingly, it is possible that ethnic identity did not significantly affect mental health.

## 5. Limitations and Prospects

This study has several limitations. First, the cross-sectional design limits the possibility of delineating causality among the variables. Therefore, a longitudinal design is needed in future research to fully explore the relationships examined in our study. Second, data were collected from two cities in south-western China, which makes it impossible to generalise the study’s findings. As an antidote, future studies should form more diverse samples from more extensive regions. Third, the data in our study were self-reported by adolescents. Considering the sensitive nature of the topic of discrimination, future studies should collect information from multiple sources, including peers and parents. Furthermore, because adolescents from different ethnic groups can differ in important ways, future studies should involve more adolescents from different ethnic minority groups and examine whether perceived discrimination’s effects on their developmental outcomes differ across them.

Despite the limitations, our study has enriched the current body of literature on perceived discrimination and ethnic minority adolescents’ developmental outcomes, and has important implications for practice geared towards reducing the negative effects of perceived discrimination on adolescents’ developmental outcomes. On a theoretical level, our study has advanced understanding of the effects of perceived discrimination, self-esteem, and ethnic identity on developmental outcomes. Moreover, in distinguishing perceived discrimination based on personal characteristics and discrimination based on group membership, as well as by comparing the different impacts and mechanisms of those two kinds of discrimination on developmental outcomes, the study has provided empirical evidence of the necessity of differentiating discrimination, particularly in a sample of Bai adolescents in China. Furthermore, the findings of our study partly support social identity theory, which extends current understandings of the implications of theories in different cultural backgrounds. Added to that, our study has provided an integrated framework for future research to explore the mechanism of different types of discrimination on developmental outcomes.

For social work interventions and social policies, our study provides empirical evidence that perceived discrimination is associated with adverse outcomes for Bai adolescents’ development. Social work interventions should be developed to lessen such negative forms of discrimination at schools. Likewise, social work programmes promoting intergroup contact should be established to help adolescents to learn more about other ethnic groups’ history, culture, and traditions, thereby improving intergroup relationships and reducing discrimination in the population [1]. Social policies emphasising the equality and unity of all ethnic groups should be continuously implemented to protect the legitimate rights and interests of all ethnic minorities and to prohibit the discrimination and oppression of any ethnic group. Last, our study showed that self-esteem mediated perceived academic discrimination and developmental outcomes, that ethnic identity mediated perceived ethnic discrimination and the same outcomes, and that self-esteem and ethnic identity were both positively related to adolescents’ development. Given those results, promoting ethnic minority adolescents’ self-esteem and ethnic identity could be an effective strategy used in social work practice to reduce the negative effects of discrimination on ethnic minority adolescents’ developmental outcomes.

## 6. Conclusions

Our findings validate perceived academic discrimination’s negative impact on mental health and perceived ethnic discrimination’s negative impact on behavioural adjustment and social competence among Bai adolescents. Meanwhile, they corroborate self-esteem’s mediating role in the relationships between perceived academic discrimination and all three dimensions of developmental outcomes among adolescents (i.e., behavioural adjustment, mental health, and social competence), as well as ethnic identity’s mediating role in the relationships between their perceived academic discrimination and both behavioural adjustment and social competence. Overall, our work has deepened current understandings of how different kinds of perceived discrimination affect developmental outcomes among ethnic minority adolescents in China and extends current understandings of the implications of social identity theory in different cultural backgrounds. Moreover, it provides practical implications for policymakers and social workers in China to promote ethnic minority adolescents’ developmental outcomes through boosting their self-esteem and ethnic identity.

## Figures and Tables

**Table 1:** Demographic information of participants (*N* = 813).

Variable		Frequency (*N*)	Percentage (%)
Gender	Male	382	47.0
	Female	431	53.0
School type	Minority School	379	46.6
	Ordinary School	434	53.4
Paternal education level	Less than primary school	46	5.7
	Primary school	149	18.3
	Middle school	347	42.7
	High school or vocational school	161	19.8
	Three-year college	60	7.4
	University or more	50	6.2
Maternal education level	Less than primary school	86	10.6
	Primary school	198	24.4
	Middle school	324	39.9
	High school or vocational school	123	15.1
	Three-year college	48	5.9
	University or more	34	4.2
Paternal occupation	Unemployed workers	437	53.8
	Manual labourer or self-employed	104	12.8
	General technical personnel	131	16.1
	Middle-level managers or professional personnel	74	9.1
	Senior managers	67	8.2
Maternal occupation	Unemployed workers	526	64.7
	Manual labourer or self-employed	61	7.5
	General technical personnel	124	15.3
	Middle-level managers orprofessional personnel	53	6.5
	Senior managers	49	6.0
Age	Mean = 13.87 (years)	*SD* = 1.06	

Note. Minority schools refer to schools established by the Chinese government to promote the educational development in ethnic minority areas, which have unique cultural characteristics of ethnic minorities and are entitled to a series of preferential policies, such as earmarked development funds.

**Table 2:** Descriptive statistics and bivariate correlations for key variables.

	Mean	*SD*	1	2	3	4	5	6	7
1. PAD	1.931	0.879	1						
2. PED	1.217	0.480	0.306 **	1					
3. SE	3.544	0.849	−0.181 **	−0.144 **	1				
4. EI	3.772	0.831	−0.052	−0.079 *	0.348 **	1			
5. BA	4.322	0.723	−0.112 **	−0.155 **	0.201 **	0.327 **	1		
6. MH	3.440	1.089	−0.204 **	−0.100 **	0.275 **	0.078 *	0.079 *		
7. SC	4.016	0.736	−0.173 **	−0.193 **	0.365 **	0.386 **	0.329 **	0.251 **	1

Note. PAD: perceived academic discrimination, PED: perceived ethnic discrimination, SE: self-esteem, EI: ethnic identity, BA: behavioural adjustment, MH: mental health, SC: social competence. * *p* < 0.05, ** *p* < 0.01.

**Table 3:** Standardised factor loadings of observed variables on latent constructs.

Latent Construct	Observed Variable	Factor Loading
Perceived academic discrimination (PAD)	PAD1	0.372
	PAD2	0.761
	PAD3	0.762
Perceived ethnic discrimination (PED)	PED1	0.658
	PED2	0.828
	PED3	0.801
Self-esteem (SE)	SE1	0.740
	SE2	0.766
	SE3	0.687
	SE4	0.649
Ethnic identity (EI)	EI1	0.679
	EI2	0.763
	EI3	0.740
	EI4	0.555
Behavioural adjustment (BA)	BA1	0.711
	BA2	0.713
	BA3	0.482
Mental health (MH)	MH1	0.672
	MH2	0.819
	MH3	0.707
Social competence (SC)	SC1	0.726
	SC2	0.724
	SC3	0.795
	SC4	0.665
	SC5	0.404

**Table 4:** Standardised direct, indirect, and total effects.

Predictors	BA (R^2^ = 0.247)			MH (R^2^ = 0.190)			SC (R^2^ = 0.320)		
	Direct	Indirect	Total	Direct	Indirect	Total	Direct	Indirect	Total
PAD	−0.116	−0.030 *	−0.146 *	−0.228 **	−0.065 ***	−0.293 **	−0.098	−0.066 ***	−0.165 *
PED	−0.118 *	−0.040 *	−0.158 *	0.029	0.001	0.030	−0.110 *	−0.041 *	−0.150 **
SE	0.127 *	-	0.127 *	0.274 **	-	0.274 **	0.278 **	-	0.278 **
EI	0.392 **	-	0.392 **	−0.013	-	−0.013	0.397 **	-	0.397 **
Age	0.033	-	0.033	−0.115 **	-	−0.115 **	−0.087 *	-	−0.087 *
Gen	−0.081 *	-	−0.081 *	0.162 **	-	0.162 **	0.041	-	0.041
SES	−0.048	-	−0.048	−0.041	-	−0.041	0.087 *	-	0.087 *
ST	0.073 *	-	0.073 *	−0.075 *	-	−0.075 *	0.017	-	0.017

Note. PAD: perceived academic discrimination, PED: perceived ethnic discrimination, SE: self-esteem, EI: ethnic identity, BA: behavioural adjustment, MH: mental health, SC: social competence, Gen: gender, SES: socioeconomic status, ST: school type. * *p* < 0.05, ** *p* < 0.01,*** *p* < 0.001.

## Data Availability

The datasets generated during and/or analysed during the current study are not publicly available due to datasets containing information that could compromise the privacy of research participants. The data that support the findings of this study are available from the corresponding author (L.Z.) upon reasonable request.
